# A Novel *wx2* Gene of *Toxoplasma gondii* Inhibits the Parasitic Invasion and Proliferation *in vitro* and Attenuates Virulence *in vivo* via Immune Response Modulation

**DOI:** 10.3389/fmicb.2020.00399

**Published:** 2020-04-07

**Authors:** Zhenrong Ma, Kang Yan, Ruolan Jiang, Jie Guan, Linfei Yang, Yehong Huang, Bin Lu, Xuanwu Li, Jie Zhang, Yunfeng Chang, Xiang Wu

**Affiliations:** ^1^Department of Parasitology, Xiangya School of Basic Medicine, Central South University, Changsha, China; ^2^Department of Forensic Medicine Science, Central South University, Changsha, China

**Keywords:** *Toxoplasma gondii*, CRISPR-Cas9 system, virulence factors, gene knockout, gene functions, *wx2* gene, immune response

## Abstract

*Toxoplasma gondii* (*T. gondii*) is an obligate intracellular apicomplexan protozoan that can parasitize most warm-blooded animals and cause severe diseases in immunocompromised individuals or fetal abnormalities in pregnant woman. The treatment of toxoplamosis has been limited by effective drugs. Our previous work indicated that the novel gene *wx2* of *T. gondii* may serve as a vaccine antigen candidate. To further investigate the molecular functions of *wx2* in highly virulent *T. gondii* (RH strain), a *wx2* gene deletion mutant RH strain (KO-*wx2*) was established using CRISPR-Cas9. The phynotype of KO-*wx2* was analyzed by plaque, invasion, and replication assays *in vitro* as well as *in vivo* virulence assays. The results indicated that the targeted deletion of the *wx2* gene significantly inhibited *in vitro* parasite growth and replication in the host cells as well as attenuated parasite virulence in the mouse model. Notably, the percentage of pro-inflammatory factors of interferon gamma (IFN-γ) and interlukin-17A (IL-17A) and anti-inflammatory factor of interlukin-10 (IL-10) in the lymph nodes were upregulated in mice infected with the KO-*wx2* strain. Our data suggested that the *wx2* gene plays an important role in the process of the parasite’s life cycle and virulence in mice. In addition, it also plays an important role in the host’s immunity reaction, mainly via Th1 and Th17 cellular immunity, not Th2.

## Introduction

As a member of the phylum apicomplexa, *Toxoplasma gondii* (*T. gondii*) is an obligate intracellular protozoan capable of infecting a world-wide range of hosts, including most warm-blooded animals like humans and cats (Saad et al., 2018; Tang et al., 2019). It is estimated that about one-third of the world’s human population has been infected with *T. gondii*. Although the infection is asymptomatic in the majority of cases, it can cause severe complications and even death in immunocompromised individuals (Jiang et al., 2018). In addition, primary infection during pregnancy can lead to spontaneous abortion, stillbirth, and serious congenital complications such as brain and visual impairment. The *Toxoplasma* vaccine remains the main preventive measure (Jiang et al., 2018). However, there are no successful *Toxoplasma* vaccines that can be applied clinically. Therefore, much of current research currently focuses on identification of virulence factors and exploring potential *Toxoplasma* vaccine candidates. Although rhoptries, micronemes, dense particles, and secretory proteins (ROPs, MICs, or GRAs, respectively) have been confirmed to be the main virulence molecules, there has been no considerable breakthrough in current research regarding new virulence factors.

The invasion of *T. gondii* is closely related to its surface proteins and secreted proteins. The surface proteins and secreted proteins of *T. gondii* play important roles in the process of invading and multiplying in host cells. For example, micro-mitochondrial proteins are important secreted proteins of *T. gondii*. Micro-mitochondrial proteins such as MIC1, MIC2, and MIC3 are important adhesion molecules and play crucial roles during gliding motility and host cell invasion (Gras et al., 2017).

Previous data showed that the *wx2* gene (GenBank accession number: AY238892) is a potential DNA vaccine candidate against *T. gondii* (Wu et al., 2007). The DNA vaccine could prolong the survival time of mice against *Toxoplasma* by challenging and stimulating the host’s protective effect against *T. gondii* infection via the CD8 + CTL pathway (Wu et al., 2004). WX2 is a membrane molecule with a molecular weight of 49kDa. WX2 is identical to the partially conserved sequence of the *Toxoplasma* antigen-associated sequence family (SRS) molecule, and no corresponding homologous protein has been found in other protein databases during bioinformatics analyses. Tan et al. constructed a double epitope vaccine that exerts cellular immunity against *T. gondii* infection. Animal experiments showed that the WX2 DNA vaccine could prolong the survival time of mice challenged with virulent RH strains of *T. gondii* (Tan et al., 2008).

To explore the molecular function of the *wx2* gene, we generated a *wx2* gene deletion mutant for the RH stain (KO-*wx2*) using CRISPR-Cas9. The phynotype of KO-*wx2* was analyzed by plaque, invasion, and replication virulence assays *in vitro* and *in vivo*. The results indicated that targeted deletion of the *wx2* gene significantly inhibited *in vitro* parasite growth and replication in the host cells and attenuated parasite virulence in the mouse model. Notably, the percentage of pro-inflammatory cytokines interferon gamma (IFN-γ) and interlukin-17A (IL-17A) and anti-inflammatory factor of interlukin-10 (IL-10) in the lymph nodes were upregulated in mice infected with the KO-*wx2* strain. Our data suggested that the *wx2* gene plays a key role in the development of the parasite’s life cycle and virulence in mice. In addition, it also plays a role in the host’s immunity reaction, mainly via Th1 and Th17 cellular immunity response, not Th2.

## Materials and Methods

### *Toxoplasma* and Host Cell Culture

Tachyzoites of *T. gondii* RH strain (type I) were cultured in human foreskin fibroblasts (HFF) using Dulbecco’s Modified Eagle medium (DMEM) supplemented with 2% fetal bovine serum (FBS). HFF cells were grown in culture flasks containing DMEM supplemented with 10% FBS, in a 37°C and 5% CO_2_ incubator.

### Construction of *wx2* Knockout *T. gondii* Strains

The *wx2* gene knockout strains were constructed as described in the literature using CRISPR-Cas9 (Wang et al., 2016, 2017). SgRNA of *wx2* was briefly transmitted into pSAG1:CAS9-U6:sgUPRT by PCR using the Q5 Mutagenesis Kit, and positive plasmid of pSAG1:CAS9-U6:sgwx2 was extracted using Endo-Free Plasmid DNA Mini Kit protocols. The resistance cassettes (DHFR^∗^-Ts) were amplified from the plasmid pUPRT-DHFR-D by PCR reaction and purified by agarose gel electrophoresis. About 20 μg positive plasmids and 2 μg purified DHFR^∗^-Ts amplicons were cotransfected into freshly harvested *T. gondii* RH tachyzoites by electroporation. The transgenic parasites were obtained by selection via 3 μM pyrimethamine (Wang et al., 2016) and PCR was performed with genome DNA as template to confirm the gene of *wx2* was knocked out. All plasmids and primers used in this study are listed in the Supplementary Table 1.

### RT-PCR

The *wx2* gene was confirmed to absolute deletion by reverse transcription PCR (RT-PCR) at mRNA level. Total RNA was extracted from tachyzoites of wild type (WT), or *wx2-*deletion mutant *T. gondii* strain, using TRIzol (Invitrogen, Carlsbad, CA, United States) according to the manufacturer’s recommendations. Reverse transcription was performed using a PrimeScript^TM^ 1st Strand cDNA Synthesis Kit. cDNAs from the KO-*wx2* and WT RH strain were tested by RT-PCR to amplify using KO-*wx2* primers listed in the Supplementary Table 1.

### Western Blotting

About 10^7^ freshly harvested tachyzoites of KO-*wx2* and RH strains were centrifuged at 3,000 rpm for 10 min. The supernatant was discarded and the precipitate was resuspended in 100 μl PBS. The samples were frozen and thawed three times and crushed for 3 min with ultrasonic pulverizer (Ningbo Scientz Biotechnology Co., Ltd., Zhejiang, China). The mixture was centrifuged at 12,000 rpm for 30 min at 4°C to obtain the supernatant. The protein concentration was measured using a bicinchoninic acid protein assay kit (Beyotime Biotechnology, Inc., Shanghai, China). A total of 30 μg proteins of RH and KO-*wx2* were boiled with SDS-PAGE sample loading buffer and added to SDS-PAGE for electrophoresis. The proteins were blotted onto a nitrocellulose membrane and blocked with 5% non-fat milk for 1 h at room temperature. Subsequently, the membrane was incubated with mouse anti-*wx2* monoclonal antibodies and anti-GAPDH monoclonal antibodies (diluted 1:2000 in TBST) at 4°C overnight. The membrane was subsequently incubated for 1 h at room temperature with the secondary antibody (HRP-conjugated rabbit anti-mouse IgG). The membrane was analyzed using an ECL western blotting system.

### Plaque Assays

The growth rates of *wx2*-deficient and WT RH strain were determined in HFF cells. When cells were grown to a sufficient density in six-well cell culture plates, 200 fresh parasites from infected mice were added into HFF cells and incubated undisturbedly at 37°C in 5% CO_2_ for 7 days. The infected HFF cells were fixed with 100% methanol (pH 7.4) for 15 min at room temperature. The fixed cells were stained with a crystal violet solution (0.2%, pH 7.0) for 10 min at ambient temperature to visualize the size of plaques by microscopy (Wang et al., 2016). This experiment was repeated at least three times and each experiment was performed in triplicate.

### Invasion Assay

About 10^6^ freshly harvested tachyzoites from mice were added to HFF cells in a 24-well plate and allowed to invade host cells for 3 h. After washing five times with PBS, the cells were fixed with 100% methanol for 5 min. The fixed cells were incubated with Giemsa dye solution (1:10, pH 6.8–7.0) for 25 min to visualize the results under microscopy (Wang et al., 2016). At least 100 cells were randomly selected to calculate the number of tachyzoites, and the wild type and knockout strains were compared using this data. The data was presented as the mean ± SD from three independent experiments.

### Proliferation Assays

About 10^6^ freshly harvested tachyzoites from mice were added to HFF cells in a 24-well plate. 4 h later, remaining free parasites were removed by three washes with PBS and cells were cultured with normal medium. After an additional 20 and 44 h incubation, the cells were fixed with 100% methanol for 5 min. Fixed cells were incubated with the Giemsa stain solution (1:10 pH 6.8–7.0) for 25 min before microscopic examination (Wang et al., 2016). Parasitic vacuoles of at least 100 random selected cells were scored. The data was expressed as the mean ± SD from three independent experiments.

### Virulence Assay

Specific-pathogen-free (SPF) inbred female KM mice (8 weeks old) were purchased from the Center of Laboratory of Animals, Central South University, Hunan, China. All mice were handled in strict accordance with guidelines of the People’s Republic of China and university’s ethics committee. Each mouse was injected intraperitoneally with 5,000 freshly harvested tachyzoites of *wx2-*deficient or WT RH strain (eight mice per strain). All mice were monitored daily until death.

### Flow Cytometry Analysis

Each Balb/c mouse was injected intraperitoneally with 2,000 tachyzoites of *wx2*-deficient or WT RH parasites. The infected mice were euthanized by cervical dislocation about 6–7 days before death. The lymph nodes were isolated, and a single cell suspension was isolated by grinding through a 40 micron sterile nylon filter. These cells were then cultured in RPMI 1640 supplemented with 10% FBS. The cells were stimulated with Leukocyte Activation Cocktail (BD PharMingen, San Jose, CA, United States) via incubation in 5% CO_2_ for 4 h at 37°C and then stained for surface and intercellular markers. The cells were resuspended in PBS and incubated with FITC-rat-anti-mouse CD4 antibody for 30 min in the dark. Fixation/permeabilization solution was added to the resuspended cells and allowed to incubate for 30 min, and the cells were washed again. Percp-cy5, 5-Rat-anti-mouse IL-17A, and PE-cy7-rat-anti-mouse IFN-γ antibodies were stained for intracellular cytokines respectively, then washed and resuspended again for flow cytometry analysis. Data were taken from all three separate experiments.

### Cytokine Detection *in vitro*

RAW264.7 cells were cultured in 6-well plates with special medium. When the cells were in the well state, about 10^6^ fresh tachyzoites from mice of KO-*wx2* and RH strains were added to the cells to invade for 24 h. Cells were collected to extract total RNA with TRIzol, and related pro-inflammatory and anti-inflammatory factors were detected by qRT-PCR. All primers are listed in the Supplementary Table 4.

### Treatment of Mice With Plasmid pEGFP-N1-WX2

Balb/c mice were infected with 2,000 *T. gondii* tachyzoites of either RH or KO*-wx2* strains, treated or not treated intraperitoneally (i.p.) with pEGFP-N1-WX2 plasmid at a dose of 10 μg/mouse every day from 1 day before infection to 5 days post-infection, and euthanized before death. Lymph nodes were isolated, single-cell suspensions were prepared, and total RNA with TRIzol were extracted after centrifugation; similarly, related pro-inflammatory and anti-inflammatory factors were detected by qRT-PCR. All primers are listed in the Supplementary Table 4.

### Plasmid Transfection

The bacterial strains pEGFP-N1-WX2 and pEGFP-N1 were inoculated into LB medium containing kanamycin antibiotic at a ratio of 1:1,000. The medium was shaken overnight at 37°C and 250 rpm, and the plasmid was extracted according to the sterile endotoxin-free plasmid extraction kit instructions. The pEGFP-N1 and pEGFP-N1-WX2 plasmids were separately transfected into 293T cells according to the attractene transfection reagent instructions. Transfected cells were cultured at a 37°C, 5% CO_2_ atmosphere for 48 h and the fluorescence expression was observed under a fluorescence microscope.

### Immunoprecipitation and LC-MS/MS Analysis

293T cells were grown to approximately 80% confluency. Plasmids of pEGFP-N1-wx2 and pEGFP-N1 plasmids were separately transfected into the 293T cells. After 48 h, the cell lysates were collected and 1 μg anti-GFP monoclonal antibody was added and incubated overnight at 4°C. 20 μl proteinG PLUS/ProteinA-Agarose beads were added to the antigen-antibody mixture and incubated for 1 h at room temperature. The suspension was centrifuged at 4°C and 2,500 rpm for 5 min, and washed three times with RIPA. The supernatant was collected for SDS-PAGE. The beads were eluted using the glycine elution buffer. The results were observed directly by silver staining and the differential protein bands were used to synthesize a sample for LC-MS/MS analysis.

### Silver Staining

After electrophoresis, silver staining fixative A was added to the separation gel and shaken slowly for 30 min at room temperature. Solution B was added and shaken for 10 min at room temperature. We then added silver staining sensitizing solution for 1 min and washed twice with water for 1 min each time. Next, the silver nitrate solution was added and shaken gently for 20 min in the dark. The silver staining solution was added and shaken until bands were clearly visible. Finally, the silver dyeing stop solution was immediately added and shaken slowly for 30 min when the band color was appropriate.

### Gene Ontology Analysis

Candidate proteins, identified from the LC-MS/MS results, were functionally annotated via the online gene function annotation tool WebGestalt^1^ using overrepresentation enrichment analysis.

### Statistical Analysis

All experiments were performed at least in triplicate. GraphPad Prism 8 was used for statistical analyses. The differences between groups were analyzed using the *t*-test, and values of *P* < 0.05 were considered statistically significant. All the results were presented as mean ± SD, which represents a summary of the data from at least three experiments. Statistics symbols used are: ^∗^*P* < 0.05, ^∗∗^*P* < 0.01, ^∗∗∗^*P* < 0.001.

## Results

### Generation and Verification of *wx2* Knockout Parasite

**FIGURE 1:**
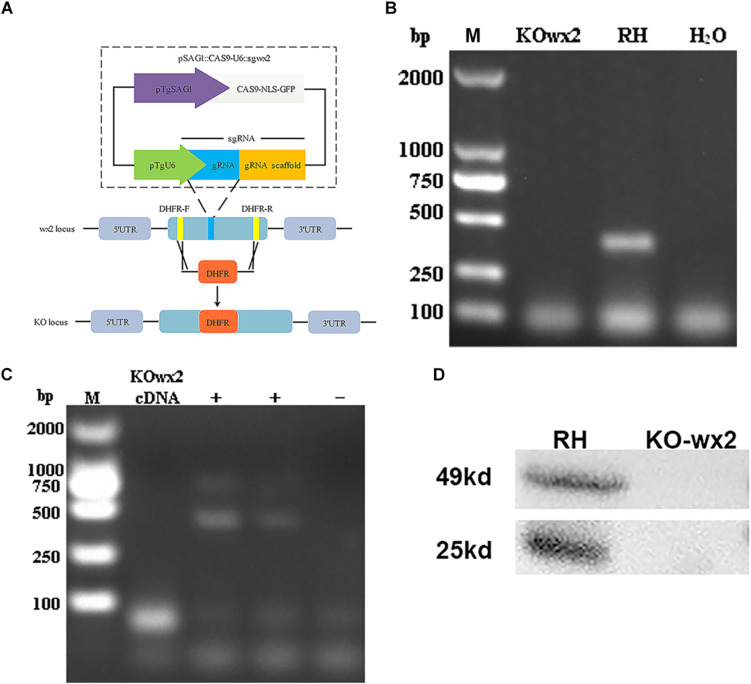
*Toxoplasma gondii wx2* gene knockout (KO) mutant construction. **(A)** Schematic illustrating CRISPR-Cas9-mediated gene knockout by insertion of the pyrimethamine-resistant DHFR^∗^-Ts in the *wx2* coding sequence. **(B)** KO forward and KO reverse primers were used to amplify the small fragment with short extension times. Diagnostic PCR demonstrated DHFR^∗^-Ts integration and *wx2* gene disruption in a representative clone compared with the wild-type RH strain. **(C)** RT-PCR verification of *wx2* gene knockout. Small fragments around the insertion site for the *wx2* gene were not detected by RT-PCR, demonstrating that the coding sequences were disrupted. **(D)** Detection of *wx2* gene expression in RH and KO-*wx2* strains at the protein level by western blotting. The results indicate that the *wx2* gene was completely knocked out.

We disrupted the *wx2* gene by insertion of DHFR^∗^ at the guide RNA-targeted region in the endogenous *wx2* locus using the CRISPR-Cas9 system (Figure 1A). Diagnostic PCR yielded the expected small fragments in the WT RH strain, while a large fragment containing about 3.3 kb DHFR-Ts was not amplified in the KO strain with a 30s extension time (Figure 1B). Additionally, the *wx2* gene was not detected in the KO strain by RT-PCR at mRNA level (Figure 1C). The western blotting results indicated that the wx2 protein was not expressed in the knockout strain, while it was detected in the wild-type strain. These results indicate that the *wx2* gene was successfully knocked out. In addition, the *wx2* molecule is a dimer and specific bands appeared around 23kd and 50kd in the WT RH strain only (Figure 1D).

### Deletion of the *wx2* Gene Affected Parasite Growth, Invasion, and Proliferation *in vitro*

**FIGURE 2:**
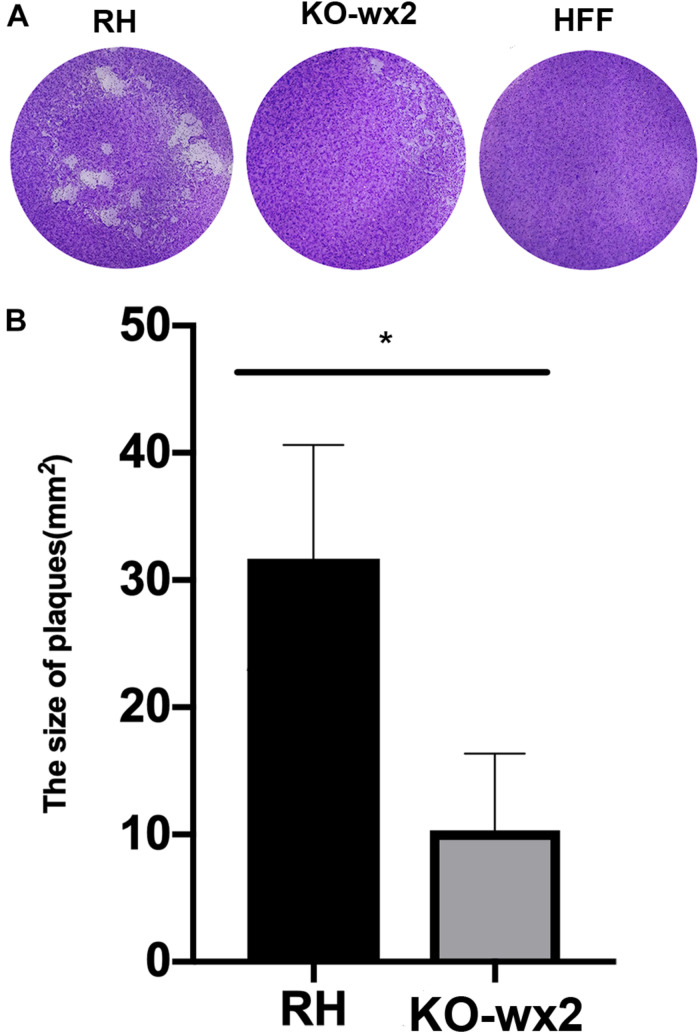
*In vitro* plaque assays. **(A)** 200 freshly harvested *T. gondii* tachyzoites of WT RH strains and *wx2*-deficient RH strains per well were added to monolayers of HFF cells in 6-well culture plates. After 7 days, the size of plaques caused by parasite proliferation was counted using a microscope. **(B)** Statistical analysis chart of A. Data are shown as the means ± SEM (three independent experiments) of each group (*n* = 3). Statistical analysis was performed by *t*-test.* *p* < 0.05.

Plaque assays were performed to analyze whether the KO-*wx2* strain has an altered lytic cycle. The plaque area were different between the RH and KO-*wx2* strains (Figure 2A), while the KO-*wx2* strain parasite displayed weakened plaque formation compared with the RH strain. The average area of plaques in the KO-*wx2* strain and WT strain were 10.3 and 31.7 mm^2^, respectively. The size of the plaques between the KO-*wx2* strain and the WT strain were statistically significantly different (*P* < 0.05) (Figure 2B). These results showed that the KO-*wx2* strain was obviously defective in the lytic cycle.

**FIGURE 3:**
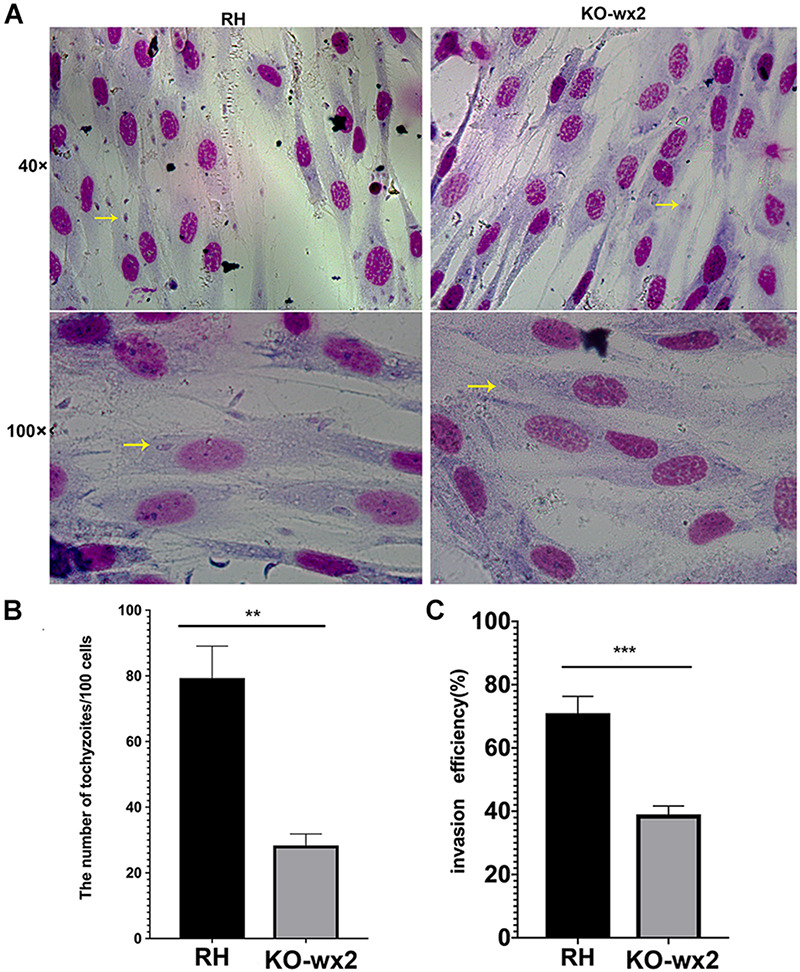
*In vitro* invasion assay. 10^6^ freshly harvested tachyzoites were added to HFF cells in a 24-well plate and allowed to invade host cells for 3 h to determine the invasion rate of the WT RH and KO-*wx2* strains. **(A)** Observing the invasion of KO-*wx2* and RH strain in HFF cells stained with Giemsa dye solution, at microscope 40× and 100×. **(B)** Statistical analysis of the number of tachyzoites calculated by 100 HFF cells in at least five fields. **(C)** Statistical analysis of the number of cells infected with tachyzoites per 100 cells in at least five fields. Data are shown as the means ± SEM (three independent experiments) of each group (*n* = 3). Statistical analysis was performed by *t*-test. ** *P* < 0.01, *** *P* < 0.001.

To further the study, we performed invasion and replication assays whether the KO-wx2 strain played a different role compared to the wild-type RH strain. Fresh parasites from mice were inoculated onto HFF cells for 3 h to investigate the invasion abilities of two different parasite strains. The number of parasites were counted in 100 random cells. Our results showed that the invasive ability was decreased in the KO-*wx2* strain. The mean number of intracellular parasites of the KO-*wx2* strain and the wild-type strain were counted at 28 and 79 per 100 cells, respectively (Figures 3A,B). The invasive rate of the KO-*wx2* strain and the wild strain was significantly different (*P* < 0.001) (Figure 3C), indicating that the *wx2* gene was involved in the process of *T. gondii* invasion into host cells.

**FIGURE 4:**
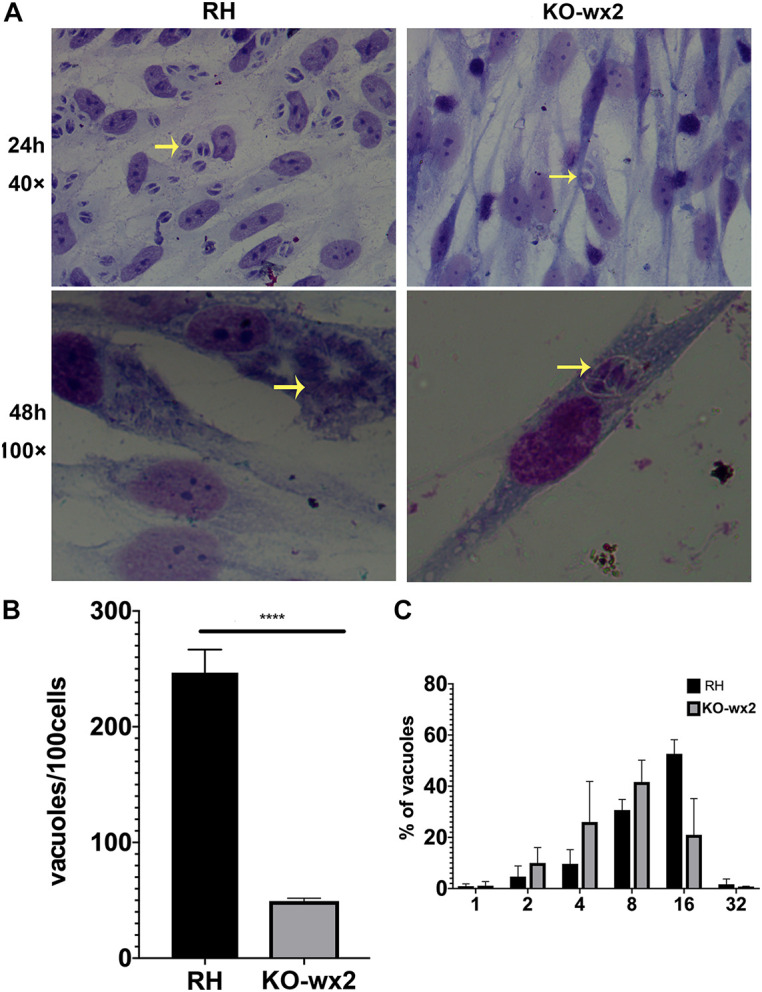
*In vitro* proliferation assays. 10^6^ freshly harvested tachyzoites were added to HFF cells in a 24-well plate and allowed to invade host cells for 24 and 48 h. Intracellular replication of the wild-type RH strain and KO-*wx2* strain were performed by scoring the number of parasites per vacuole and total vacuoles per 100 cells. **(A)** Observing the intracellular replication of KO-*wx2* and RH strains in HFF cells stained with Giemsa dye solution at microscope 40× (24 h) and 100× (48 h). **(B)** Statistical analysis of the total vacuoles calculated by 100 HFF cells randomly (24 h assay). **(C)** Statistical analysis of the number of parasites per vacuole calculated by 100 HFF cells randomly (48 h). Data are shown as the means ± SEM (three independent experiments) of each group (*n* = 3). Statistical analysis was performed by *t*-test. ****P < 0.0001.

Similarly, in the proliferation assay, KO-*wx2* strain and the wild-type RH strain were allowed to invade cells for 24 and 48 h. After 24 h, at least 100 cells were randomly selected to calculate the number of vacuoles. There was a significant difference (*P* < 0.001) in the number of vacuoles formed by the two different strains in HFF cells, and the mean number of vacuoles in the RH strain (247) was greater than in the KO-*wx2* strain (49) (Figures 4A,B). After 48 h, the number of parasites per vacuole was calculated by observing 100 random HFF. The results indicated that the majority of the vacuoles formed by the KO-*wx2* strain contained eight parasites, while the RH strain contained 16 parasites (Figure 4C). These findings showed that infection of HFF cells with the KO-*wx2* strain leads to a significant reduction in parasite multiplication and indicated that the *wx2* gene is required for the parasite’s ability to invade and reproduce within the host.

### *wx2* Gene Deletion Decreased Parasite Virulence in Mice

**FIGURE 5:**
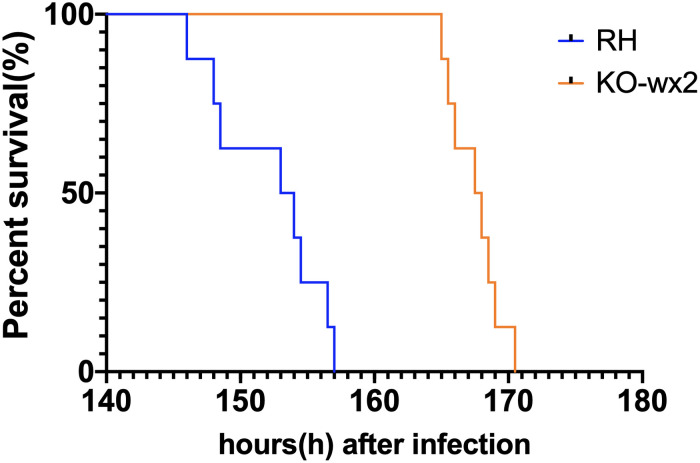
Survival curves of KM mice infected with *T. gondii* wild-type strain (RH) or KO-*wx2* strain. Each group was composed of eight mice and the two groups of mice were challenged with 2,000 tachyzoites. Every mouse was monitored daily until death.

We tested whether knock out of the *wx2* gene in *T. gondii* RH strain would change parasite virulence. 2,000 fresh WT RH tachyzoites and an equal number of KO-*wx2* tachyzoites from mice were used to infect KM mice. The result showed that the mice of the RH group died within 6–7 days after infection, while mice in the KO group clearly survived for a little longer. The survival times of the two groups were statistically different (*P* < 0.05) (Figure 5). The experimental results showed that deletion of the *wx2* gene had an effect on the virulence of *T. gondii* in mice, which could delay the death of the mice. This suggests that the *wx2* gene was an essential virulence factor of *T. gondii*.

### KO-*wx2* Strain Infection Induced Stronger Immune Response in Mice Model

**FIGURE 6:**
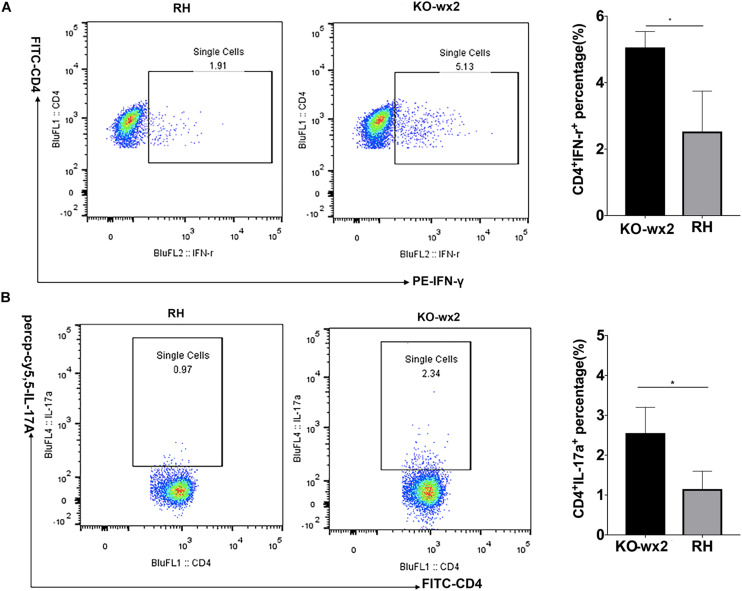
KO-*wx2* strain could cause a stronger inflammatory response in mice. Mice were euthanized and their lymph nodes of wild-type strain (RH) or KO-*wx2* strain were isolated. Lymph nodes were stained with anti-mouse CD4, IL-17A, and IFN-γ mAbs. The percentages of CD4 + IFN-γ + **(A)** and CD4 + IL-17A + **(B)** were analyzed by FACS (left), and the results of the statistical analysis are shown (right). Data are shown as the means ± SEM (three independent experiments) of each group (*n* = 3). Statistical analysis was performed by *t*-test. * *P* < 0.05.

The mice were euthanized about 6–7 days after infection with RH and KO-*wx2* strains. The lymph nodes of each group were removed, and the expression of IFN-γ and IL-17 were detected by flow cytometry. Compared to the RH group, the levels of pro-inflammatory IFN-γ were significantly increased in the lymph nodes of mice infected with KO-*wx2* strain (*P* < 0.05). The mean of the CD4^+^IFN-γ ^+^ percentage of KO-*wx2* strain and RH strain were 5.060 and 2.530, respectively (Figure 6A). Similarly, the Th17 cytokine level was strikingly increased in the KO-*wx2* group. The mean CD4^+^IL-17A ^+^ percentage of KO-*wx2* strain was 2.553, obviously higher than in the RH strain (1.15). The difference was statistically significant (*P* < 0.05) (Figure 6B). In addition, the relative expression of pro-inflammatory factors (IL-17A, IFN-γ) and anti-inflammatory factors of IL-10, transforming growth factor-β (TGF-β), and interlukin-4 (IL-4) in mice lymph nodes were detected by qRT-PCR. The results showed that related pro-inflammatory (IL-17A, IFN-γ) and anti-inflammatory factors IL-10 were higher in KO-*wx2* strain, and the difference between KO-*wx2* and RH strain were statistically significant (*P* < 0.05). There were no statistically significant differences between KO-*wx2* group and RH strain group at the expression level of TGF-β and IL-4 (see Supplementary Figure 1).

### KO-*wx2* Strain Infection Induced Stronger Immune Response *in vitro*

**FIGURE 7:**
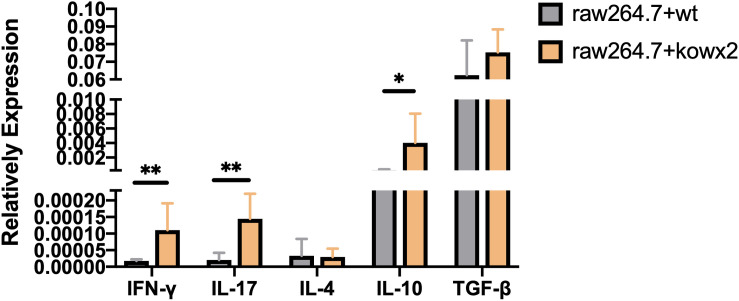
Related cytokines detection by qPCR in RAW264.7 macrophages infected by RH strains and KO-wx2 strains. Data presented are the mean ± SD from three independent experiments. Statistical analysis was performed by *t*-test. * *P* < 0.05, ** *P* < 0.01.

Cytokine detection in infected RAW264.7 cells showed that the related pro-inflammatory (IL-17A, IFN-γ) and anti-inflammatory factor, including IL-10, were higher in RAW264.7 cells infected with the KO-*wx2* strain. There was a statistically significant difference of IL-17A (*P* < 0.01), IFN-γ (*P* < 0.01), and IL-10 (*P* < 0.05) expression in cells infected with the RH and KO-*wx2* strain. There was no statistical difference in TGF-β and IL-4 level between cells infected with the RH and KO-*wx2* strain (Figure 7).

### Inflammation-Related Factors Change With pEGFP-N1-WX2 Plasmid Intervention *in vivo*

The results showed that before plasmid pEGFP-N1-WX2 intervention, the relative expression of IL-17A, IFN-γ, and IL-10 within the group of mice infected with the KO-*wx2* strain was higher than the RH strain group. This data was statistically different(*P* < 0.05). After plasmid pEGFP-N1-WX2 intervention, compared to the RH strain group, except for TGF-β and IL-4, the relative expression of IL-17A, IFN-γ and IL-10 in KO-*wx2* plus plasmid group was decreased. The data were statistically different (*P* < 0.05; see Supplementary Figure 1). This data further proved that *wx2* was related to virulence of *T. gondii*.

### KEGG Pathway and Gene Ontology Analysis

**FIGURE 8:**
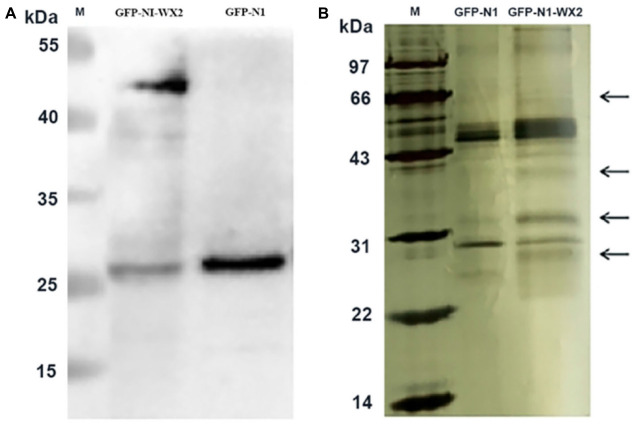
Immunoprecipitation silver staining analysis. **(A)** Western blot analysis of *wx2* plasmid expression in 293T cells. **(B)** Identification of GFP-wx2 plasmid by immunoprecipitation electrophoresis silver staining.

**FIGURE 9:**
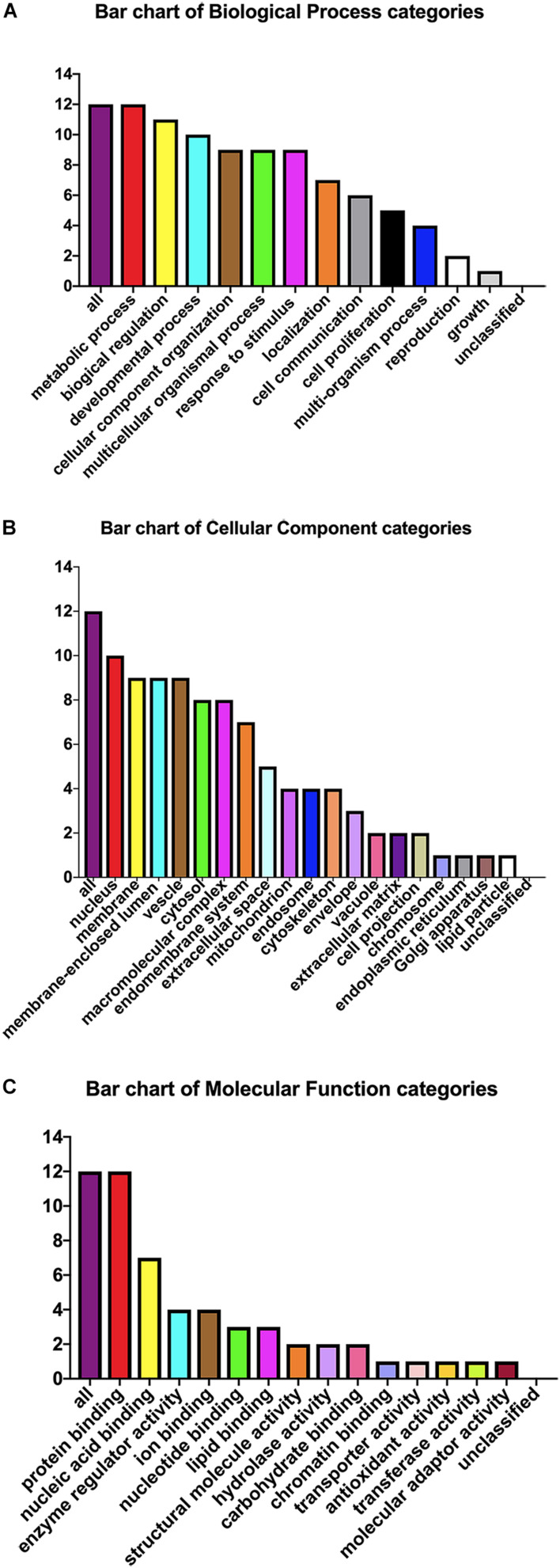
Gene ontology analysis. Candidate protein distribution of gene ontology terms at level 3 for: **(A)** Biological Process, **(B)** Cellular Component, **(C)** Molecular Function categories.

The pEGFP-N1-WX2 plasmid was transfected into 293T cells for 48 h, then the lysate was collected. Part of the lysate was used for immunoblot analysis and the results showed that the GFP protein band size was about 27 kd in the control group. There were two bands in the experimental group, 27 and 50 kd, consistent with expected GFP protein and GFP-wx2 protein sizes (Figure 8A). The silver staining results showed that there were four differentially expressed bands in the experimental group compared to the control group. The molecular weights were between 22–30, 31–40, 40–43, and 60–70 kd (Figure 8B). Four different protein bands from the experimental group were combined into one sample for LC-MS/MS analysis. A total of 29 candidate proteins were selected. The top ten LC-MS/MS results are listed in Supplementary Table 2. Only 12 candidate proteins were annotated with the online gene functional annotation tool WebGestalt. The obtained annotated proteins were concentrated mainly in the biological processes of metabolism, biological regulation, developmental process, cellular component organization, multicellular organismal process, and response to stimulus (Figure 9A). Cellular component analysis included nucleus, membrane, membrane-enclosed lumen, vesicle, cytosol, and macromolecular complex processes (Figure 9B). Their molecular functions were focused mostly on binding abilities including protein binding, nucleic acid binding, ion binding, and enzyme regulator activity processes (Figure 9C). The remaining lysate was collected for KEGG pathway analysis, and the results showed that the MAPK signaling pathway and p53 signaling pathway was most significant. In the gene ontology analysis of *wx2*, the downregulated genes included GADD45A and DDIT3, while the upregulated genes included RAC1 and PPP3 (see Supplementary Table 3).

## Discussion

*ToxoplasmaT. gondii* is an opportunistic pathogen, and the infection can cause serious health problems and become life-threatening in immunocompromised individuals. Although death caused by toxoplasmosis is rare, clinical cases of encephalitis or ocular toxoplasmosis are common (Paul et al., 2018). Repeated lytic cycles of invasion, replication, and egress drive both the propagation and the virulence of this parasite. The research currently has focused mainly on rhoptries, micronemes, and dense particles, and these secretory proteins that have been shown to be closely related to the invasion, proliferation, export, and virulence of *T. gondii* (Hunter and Sibley, 2012). Hence, it is more important to discover new virulence-related molecules of *T. gondii* and provide a basis for future treatment of toxoplasmosis by functional exploration.

Our previous research has shown that WX2 was a membrane molecule and the *wx2* gene was a dependable DNA vaccine candidate of *T. gondii* (Wu et al., 2007). In further studies, we found that the MAPK signaling pathway and the p53 signaling pathway were most significantly different by differential gene pathway enrichment analysis. Gene ontology analysis of *wx2* showed that the downregulated genes included GADD45A and DDIT3, and the upregulated genes included RAC1 and PPP3 (Supplementary Table 3). GADD45A may play an important role in cell adhesion, migration, and invasion ability. Thus, downregulated GADD45A could exert a promoting role on cytoskeletal stability and cell proliferation. DNA damage-inducing transcript (DDIT3) has a strong relationship with apoptosis. Therefore, we hypothesized that the function of the *wx2* gene may be involved in maintaining cytoskeletal stability, promoting cell proliferation, and regulating apoptosis. It has been reported that expression of the RAC1 gene reduced cell migration through the p38MASK phosphorylation-mediated MASK signaling pathway (Zimmermann et al., 2008).Therefore, we speculated that the *wx2* gene may also have a similar effect on inhibiting cell migration. Unfortunately, these conjectures have not been confirmed. The biological process analysis of obtained annotated proteins concentrated on metabolism and biological regulation; it was revealed that the *wx2* gene may be involved in the growth and reproduction of *T. gondii*, thereby affecting its virulence. Cellular component analysis included the nucleus, membrane, membrane-enclosed lumen, vesicle, and cytosol. This data suggested that the *wx2* gene may have a regulatory effect on maintaining the normal structure of *T. gondii* and on its genetics and metabolism function. Their molecular functions were focused mostly on binding ability, including protein binding, nucleic acid binding, ion binding, and enzyme regulator activity processes, and hinted that *wx2* has great potential as a drug and vaccine target.

To investigate the function of the *wx2* gene, we successfully used the CRISPR-Cas9 system to create the KO-*wx2* strain in this study. The findings of this study indicated that deletion of *wx2* gene affected parasite growth, invasion and proliferation *in vitro*, and affected virulence in mice. We found that the virulence of KO-*wx2* parasites was not completely eliminated and only had a significant delay in host survival time. Compared with wild strains, the invasion rate of KO-*wx2* strain is lower and it reproduces a bit slower, so its virulence is weakened to prolong host survival. In addition, only the RH strain of the *wx2* gene was studied, and the function of the *wx2* gene in other strains has not been confirmed. Further research is needed to confirm *wx2* gene function in other *T. gondii* strains.

It is interesting to note that both pro-inflammatory factors of IFN-γ and IL-17A were significantly upregulated in mouse models infected with KO-*wx2* strain, as these cytokines are associated with Th1 and Th17 immunity, respectively. Both *in vivo* and *in vitro* experiments can prove that there was no statistical difference in the expression of IL-4 between the RH and KO-*wx2* groups. It can be explained that Th2 type cellular immunity has little effect on the immune regulation of *T. gondii* after knocking out the *wx2* gene during the acute infection phase. Moreover, the anti-inflammatory factor IL-10 was higher in mice infected with the KO-*wx2* strain. In addition, the relative expression of both pro-inflammatory (IFN-γ and IL-17A) and anti-inflammatory (IL-10) factors were highly decreased in the KO-*wx2* + plasmid group after plasmid pEGFP-N1-WX2 intervention. This result further indicated that *wx2* gene was related to the virulence of *T. gondii*. The cytokine kinetics profile change during infection will be observed in a future study.

IL-10 is an essential anti-inflammatory cytokine that plays important roles as a negative regulator of immune responses to microbial antigens, and IL-10-mediated STAT3 has an anti-inflammatory effect (Butcher et al., 2005). IL-10 is also critical in many infections that could trigger an adaptive immune response. For example, during *T. gondii* infection, IL-10 produced by Th1 cells played a crucial role in limitting otherwise excessive Th1 cell response (Jankovic et al., 2007; Lang et al., 2013). Although IFN-γ secretion is essential for control of most intracellular pathogens, host survival also depends on the expression of IL-10 during both the acute and chronic phases of *T. gondii* infection. IL-10 not only exerts a regulatory role on *T. gondii* infection, but also plays an important role in stimulating infected macrophages to release NO and mediate intracellular killing of the parasite (Jankovic et al., 2007). In our experiments, loss of the *wx2* gene led to higher levels of IL-10, indicating that the IL-10 may have a protective effect and prolong the survival time of mice.

TGF-β is a broadly immune suppressive mediator, which plays a crucial role in immunological tolerance. Parasites residing in the mammalian host for a long time are often associated with both generalized immunosuppression and elevated TGF-β expression (Johnston et al., 2016). Thus, the changes in the levels of TGF-β are strongly related to host immunity. In our studies, it seemed that the *wx2* gene hasn’t obviously influenced TGF-β.

As is known, IFN-γ is a critical factor in host protective immunity against *Toxoplasma* infection, as it limits the spread of tachyzoites and the rate of conversion to bradyzoites (Takacs et al., 2012; Ohshima et al., 2014). Research has shown that control of *Toxoplasma* infection depends on a strong Th1 response (Takacs et al., 2012; Radke et al., 2018; Sasai et al., 2018). On the other hand, if *T. gondii* has evolved several means to evade the detrimental actions of innate and adaptive immunity during the host-parasite co-evolution process, IFN-γ plays a crucial role in the antimicrobial host immune mechanisms (Sacks and Sher, 2002; Laliberte and Carruthers, 2008; Stutz et al., 2012). *T. gondii* infection can avoid the host’s immune response by inhibiting the production of IFN-γ. Our research showed that the KO-*wx2* strain stimulated the host’s immune response then produced more IFN-γ, which could suggest that knocking out the *wx2* gene can stimulate host immune response effectively and destroy the parasites’ immunity evasion to some extent.

IL-17A, a pro-inflammatory cytokine produced by Th17 cells, contributes to multiple inflammation responses (Tajima et al., 2008; Passos et al., 2010). IL-17A participates in multiple host defenses against many pathogens, including bacteria and fungi (Saijo et al., 2010; Kumar et al., 2016; Matsuzaki and Umemura, 2018). The importance of IL-17A and the IL-17A receptor in host defense has been fully affirmed and understood. In addition, some studies reported that IL-17A has a protective effect in *T. gondii* infection (Ishigame et al., 2009; Moroda et al., 2017). IL-17A^–/–^ and IL-17AR^–/–^ mice showed increased susceptibility to *Toxoplasma* infection (Kelly et al., 2005). It was also reported that IL-17A played a positive role in host survival to *T. gondii* due to suppression of the allergic reaction induced by IFN-γ and Tg.HSP70 rather than its function on pathogen growth (Moroda et al., 2017). Th17 cells play an important role in *Toxoplasma* infection and immunity and exert a double effect. Th17 cells respond to the antigen at an earlier stage and the protective function is relatively strong in the progression of *Toxoplasma* infection when the host immune status is fit. They can effectively control and reduce the clinical symptoms of the host before pathological damage occurs. But when the host’s immunity is low or the infection is severe, Th17 can activate more immune cells (macrophages and fibroblasts), which inhibit parasite activity but also induce serious immunopathological changes in the host, turn the disease chronic, or even cause death. Th17 cells also recruit neutrophils to mobilize non-specific immune cells resistant to *Toxoplasma* infection at an early stage. However, since neutrophils are a kind of acute inflammatory cell, massive activation could cause a strong acute inflammatory response if not controlled.

We found that IL-17 and IFN-γ were simultaneously increased in mice infected with the KO*-wx2* strain and had longer survival time than mice with the RH strain. As such, we had to consider two points. One was that a stronger immune response needed to be stimulated to counterbalance the inflammatory response in a mouse model of knockout strain infection. Therefore, a stronger immune response may kill a part of *T. gondii* in the body, prolonging the survival time of the mice. Another point was that either IL-17 or IFN-γ played a protective role in the host. Recent research has shown that AMPs induced by IL-17 exert a protective function on dysbiosis during *T. gondii* infection. It indicated that the protective immune response against *T. gondii* must depend on both the Th1-IFN-γ axis and the Th17-AMP axis, respectively acting on controlling invasive pathogens and preventing dysbiosis, barrier, and microbial spread (Cervantes-Barragan et al., 2019). These results reflect that Th17 may exert protective function against *Toxoplasma* infection, although its protective mechanism is unclear and requires further confirmation.

This article was the first exploration of the genetic function of the *wx2* gene. It has a significant meaning not only in the study of *T. gondii* virulence molecules, but also in the study of *Toxoplasma* vaccine target molecules. Previous work has shown that the *wx2* gene expressed not only in the acute phase but also in the chronic phase. We hope it could be used as a drug target or vaccine candidate for use against acute and chronic *Toxoplasma* infection in the future.

## Data Availability Statement

All datasets generated for this study are included in the article/Supplementary Material.

## Ethics Statement

All experimental animals were used according to and with the approval of the local ethics committee for the use of animals at the Central South University (Changsha, China). The microorganisms included in this study were handled according to the General Biosafety Standard for Microbiological and Biomedical Laboratories of the People’s Republic of China with protocol numbers WS233-2002.

## Author Contributions

ZM and RJ conceptualized the study. ZM and KY worked on the methodology. LY, BL, and XL were responsible for the software. JG, KY, and JZ did the analysis. ZM prepared the original draft. YH participated in author’s proof. XW and YC edited the manuscript. XW was responsible for the project administration and funding acquisition.

## Conflict of Interest

The authors declare that the research was conducted in the absence of any commercial or financial relationships that could be construed as a potential conflict of interest.

## Footnotes

1 http://www.webgestalt.org/option.php
