# Corrigendum: A Novel *wx2* Gene of *Toxoplasma gondii* Inhibits the Parasitic Invasion and Proliferation *in vitro* and Attenuates Virulence *in vivo* via Immune Response Modulation

**DOI:** 10.3389/fmicb.2020.01882

**Published:** 2020-08-20

**Authors:** Zhenrong Ma, Kang Yan, Ruolan Jiang, Jie Guan, Linfei Yang, Yehong Huang, Bin Lu, Xuanwu Li, Jie Zhang, Yunfeng Chang, Xiang Wu

**Affiliations:** ^1^Department of Parasitology, Xiangya School of Basic Medicine, Central South University, Changsha, China; ^2^Department of Forensic Medicine Science, Central South University, Changsha, China

**Keywords:** *Toxoplasma gondii*, CRISPR-Cas9 system, virulence factors, gene knockout, gene functions, *wx2* gene, immune response

In the original article, there was a mistake in Figure 8A as published. The label on the picture was reversed. The corrected Figure 8 appears below.

**Figure 8:**
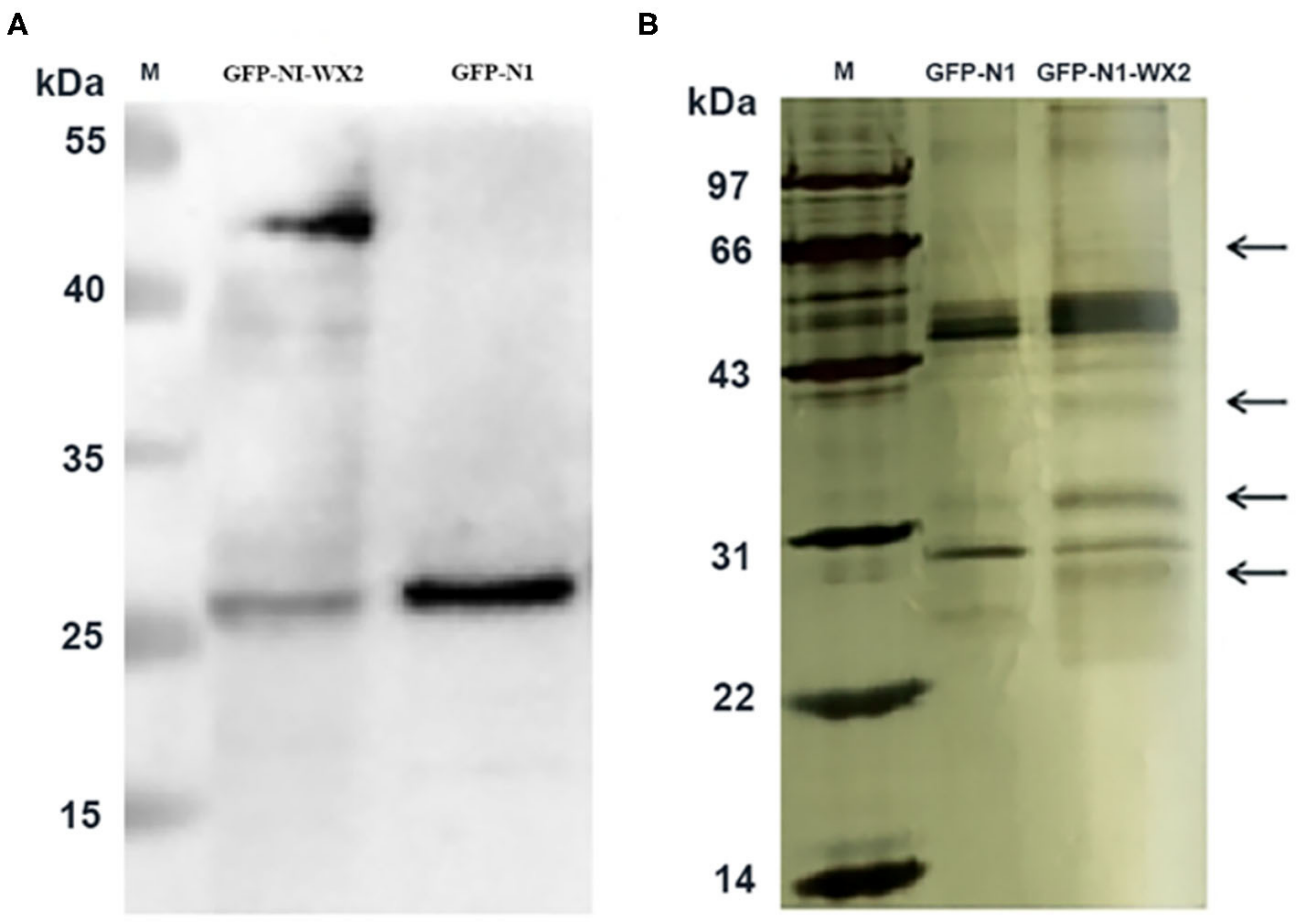
Immunoprecipitation silver staining analysis. **(A)** Western blot analysis of *wx2* plasmid expression in 293T cells. **(B)** Identification of GFP-wx2 plasmid by immunoprecipitation electrophoresis silver staining.

The authors apologize for this error and state that this does not change the scientific conclusions of the article in any way. The original article has been updated.

