# Supplementary material for: A Novel wx2 Gene of Toxoplasma gondii Inhibits the Parasitic Invasion and Proliferation in vitro and Attenuates Virulence in vivo via Immune Response Modulation
Source: Front Microbiol. 2020 Apr 7;11:399. doi: 10.3389/fmicb.2020.00399 (PMC7154108; doi:10.3389/fmicb.2020.00399)
Supplement: Supplementary file 1 [file Data_Sheet_1.pdf]

Supplementary Material

Supplementary Table 1

Primers used to construct pSAG1::Cas9-U6::sg*wx2*, *wx2*-DHFR and verify *wx2* mutant

Primer	Sequence（5'-3'）
sg*wx2*-F	GAAGAAATTCCTCCATACTCGTTTTAGAGCTAGAAATAGC
sg*wx2*-R	AACTTGACATCCCCATTTAC
wx2-DHFR-F	CAAAAACACATTCCTCACAGACGCGATGGCGTCCTGCAC
wx2-DHFR-R	ACCTCACATGGGACCCCGTGCGAGTGTTTTGAGGGTGGT
JD-*wx2*-F	GAAGAAATTCCTCCATACTC
JD-*wx2*-R	CGGACAGGTATCCGGTAAG
KO-*wx2*-F	TCCGTAAAGCGGTGAGTGTCG
KO-*wx2*-R	CGGTGTCGCCTGACTTCTGTG
RPL-F	TACGTTTTCTGTTCTGTAGATG
RPL-R	GGAAGAAGACTATTATTATGGC

Supplementary Table 2

LC-MS/MS Analysis Results (Top10)

Accession	name	Description	Score	MW(kDa)
F8VZ49	HNRNPA1	Heterogeneous nuclear ribonucleoprotein A1 (Fragment)	324.94	25.7
P04075	ALDOA	Fructose-bisphosphate aldolase A	311.01	39.4
P60709	ACTB	Actin, cytoplasmic 1	280.13	41.7
A0A087WVQ9	EEF1A1	Elongation factor 1-alpha 1	247.50	47.9
P06733	ENO1	Alpha-enolase	227.96	47.1
P10809	HSPD1	60 kDa heat shock protein, mitochondrial	214.26	61.0
E7EUT5	GAPDH	Glyceraldehyde-3-phosphate dehydrogenase	195.62	27.9
E9PKE3	HSPA8	Heat shock cognate 71 kDa protein	175.31	68.8
H0YB39	HNRNPH1	Heterogeneous nuclear ribonucleoprotein H (Fragment)	164.68	30.5
B0YJC4	VIM	Vimentin	161.57	49.6

Supplementary Table 3

Difference Genes Enrichment Analysis in Pathway

Gene Set Name	Gene Number	P-value	Gene Name
KEGG-MAPK-SIGNALING-PATHWAY	16	1.38E-05	GADD45A,JUN,HSPA1A,PRKACB,DUSPI, RAC1,FOS,SOS1,PPP3CA,MAX,HSPA1B,HSPA8,PPP3R1,DDTT3,NR4A1,NF1
BIOCARTA-PS3-PATHWAY	5	1.04E-04	GADD45A,RB1,CDKN1A,ATM,TIMP3
KEGG-STARCH-AND-SUCROSE-METABOLISM	6	2.46E-03	UGDH,AMY1A,AMY1B,AMY1C,AMY2A,AMY2B
BIOCARTA-PPARA-PATHWAY	6	3.11E-03	JUN,HSPA1A,PRKACB,DUSP1,RB1,EHHADH
BIOCARTA-BCR-PATHWAY	5	3.11E-03	JUN,RAC1,FOS,SOS1,PPP3CA
BIOCARTA-BCR-PATHWAY	4	3.11E-03	GADD45A, JUN,CDKN1A,ATM
KEGG-PATHWAY-IN-CACER	13	3.11E-03	JUN, RAC1, FOS,SOS1,MAX, RB1, CDKN1A,CDK6,PTEN,CCDC6,ITGA3,
KEGG-CELL-CYCLE	8	3.11E-03	GADD45A,RB1,CDKN1A,ATM,CDK6,CUL1,ANAPC7,STAG1
KEGG-FATTY-ACID-METABOLISM	5	3.11E-03	EHHADH,ACADM,ACSL3,ACADL,ALDH1B1

Supplementary Table 4

Primers used to qRT-PCR

IL-17A-F	GTGAGCTCCAGAAGGCCCTCAGA
IL-17A-R	GACCCTGAAAGTGAAGGGGCAGC
IFN-γ-F	GGAACTGGCAAAAGGATGGTGAC
IFN-γ-R	GCTGGACCTGTGGGTTGTTGAC
IL-10-F	AGCCGGGAAGACAATAACTG
IL-10-R	CATTTCCGATAAGGCTTGG
TGF-Beta-F	AACTATTGCTTCAGCTCCACAG
TGF-Beta-R	AGTTGGCATGGTAGCCCTTG
GAPDH-F	TGTTTCCTCGTCCCGTAGA
GAPDH-R	ATCTCCACTTTGCCACTGC

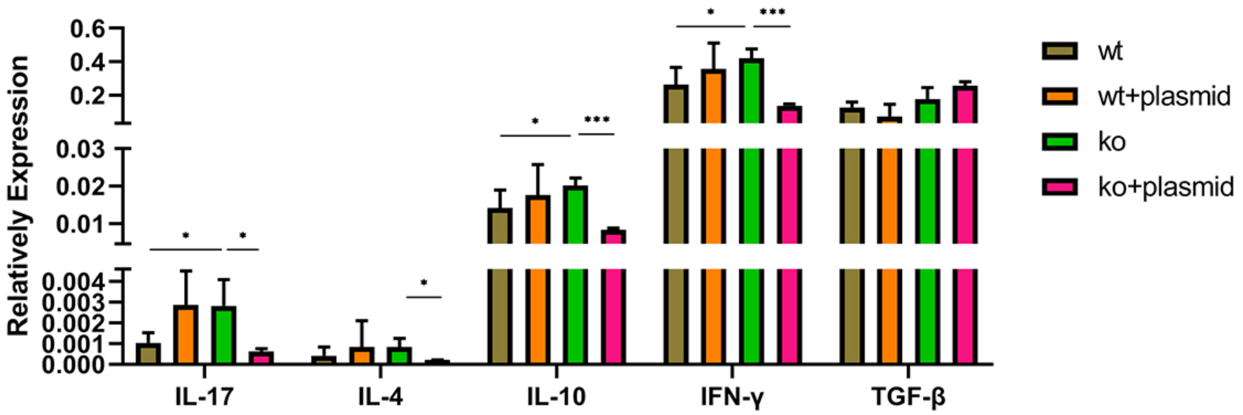

Supplementary Figure 1 Related cytokines detection by qPCR in lymph nodes cells: mRNA levels of IFN-γ, IL-17, IL-10 ,TGF-β and IL-4 in mice of RH (n=5), KO-wx2(n=5), RH+ plasmid (n=5) and KO-wx2+plasmid (n=5).The data were expressed as the mean±SD from three independent experiments. Statistical analysis was performed by t-test .* P <0.05, ** P <0.01, *** P <0.001.
